# Impact of Psycho-Educational Activities on Visual-Motor Integration, Fine Motor Skills and Name Writing among First Graders: A Kinematic Pilot Study

**DOI:** 10.3390/children7040027

**Published:** 2020-04-02

**Authors:** Livia Taverna, Marta Tremolada, Barbara Tosetto, Liliana Dozza, Zanin Scaratti Renata

**Affiliations:** 1 Faculty of Education, Free University of Bozen-Bolzano, 39042 Brixen-Bressanone, Italy; liliana.dozza@unibz.it (L.D.); Renata.Scaratti-Zanin@unibz.it (Z.S.R.); 2 Department of Development and Social Psychology and Department of Child and Woman Health, University of Padua, 35100 Padua, Italy; marta.tremolada@unipd.it; 3 Department of Women and Child’s Health, Pediatric Hematology, Oncology and Stem Cell Transplant Center, University of Padua, 35100 Padua, Italy; 4 Medical School for Health Professions, “Claudiana”, 39100 Bozen, Italy; Barbara.Tosetto@claudiana.bz.it

**Keywords:** handwriting, visuo-motor integration, fine motor skills, name writing, automaticity, handwriting fluency, automaticity, pressure

## Abstract

This pilot study presents the effects on acquisition of pre-writing skills of educational activities targeting visual-motor integration and fine motor skills on a convenient sample of first graders. After a 10-week intervention program, visual perceptual skills and fine motor control were tested on 13 six-year-old aged children. Participants completed the Beery-Buktenica VMI and the manual dexterity scale of the Movement ABC-2 at baseline (T1), after the intervention program (T2), and one month after the end of the educational activities (T3). Children’s writing pressure, frequency, and automaticity were measured using a digitizer during the administration of name writing test at T1, T2, and T3. The purpose of the study was to investigate changes in visual-perceptual abilities and fine motor skills after the intervention program and examine correlational effects on children’s kinematic writing performances. Findings reveal that educational activities impacted positively on children’s visual motor coordination component of writing improving VMI scores. No statistically significant difference was detected across the three time points on students’ manual dexterity skills. Measurement of writing kinematics allows to report and document variations in children’s writing during intervention. This pilot study discusses these findings and their implications for the field on early childhood acquisition of foundational skills for handwriting. It also proposes potential topics for future research on this field.

## 1. Introduction

Learning to write is still one of the most fundamental skills that is taught in the first cycle of education. Despite the introduction of multiple compensatory devices for writing and software/apps supporting the first literacy, or the prominent trend to introduce word-processing printed text, handwriting continues to be a “survivor” skill for children entering school. For example, we currently live in a world in which academic activity and class text production for grades 1–12 is still mainly produced by hand [1,2]. Likewise, in some countries (i.e., Italy), students are required to complete final exams for admission in higher grades with handwritten assignments. Nevertheless, learning to write still appears to be a challenge for many children who face literacy. Incidence rates for difficulties in learning to write varies from 10% to 34% of the school population [3,4], and up to 23% of them are related to the mechanics of handwriting [5].

Children avoid writing when this activity is perceived as particularly tiring as not yet fully automated, when they are unsatisfied with the quality of handwritten text because it is too messy to be read even by the author, or when they are writing so slowly that they might not be able to finish classwork or tests. Disengagement in commitment to write or loss of interest in practicing the acquisition of this competence could be a sign that the child has begun to believe that he/she is not a skilled writer [6]. Writing legibly and quickly enough to stay at the pace of school activities is required to take notes, to do the homework, and to end the evaluation tests in time [7]. Struggling in learning to write can profoundly affect a child’s development, have long-term outcomes on his/her school performance, and ultimately help define a student profile with low self-esteem that perceives himself/herself as a poor writer with limited learning skills [8].

### 1.1. Relationship between Fine Motor Skills, Eye-Hand Coordination and Writing Performance

Numerous studies have analyzed the relationship between fine motor skills (hereafter FMS) and the main predictors of writing performances. Handwriting performance has been reported to be influenced from visual perception, eye–hand coordination, and visual–motor integration [9,10,11,12]. For example, previous studies investigating the association between visual-perception and handwriting outcomes have demonstrated that visual-motor integration is a significant component of writing performance [11,13,14,15,16,17]. The visual component allows children to discriminate forms, to recognize their specific characteristics, and to identify their orientation, while the motor component, if properly developed, allows the realization of a wide range of ordered and sequential movements. Alphabet letters are legible when strokes of each grapheme are not ambiguous and the relative letters’ height is correct in order to avoid any confusion in the decoding. Writing legibly requires the child to master small hand muscles in order to form the desired traits. For this reason, FMS have been considered a necessary component in the acquisition of writing, as well as in its expression. Correlational studies in typically developing children reveal a significant association between visual perception skills and lower-case alphabet writing speed [9]. Oculo-manual coordination and text legibility are strongly associated [14,15,18] and the same is true for eye-hand coordination and fluency [19]. Children with difficulties in writing likely show very low performances at the Ayeres Motor Accuracy Test and at the Visual Motor Integration Test. Conversely, those children who perform well at VMI also exhibit better ability to reproduce legible letters [13]. Klein and colleagues [20] have examined the relationship between text legibility, fluency, and handwriting performance. Results indicated that visual-perceptual component and upper limb motor skills were significant predictor of handwriting performance with fluency in copying accounting for the 26% of variance. Indeed, delays in the development of fine motor skills have been considered the cause of poor writing in terms of readability and fluency [21,22].

Previous research has shown that writing performance can be improved if FMS [23,24] and visual-perceptive abilities are strengthened [25]. At different ages, and in relation to different schooling orders, these factors occur as predictors of writing performance. In order to form alphabet letters, placing words and letters right on the page, respecting right size and spacing, and applying the proper amount of pressure on the paper and on the writing tool, children must control and coordinate many different abilities.

### 1.2. Relationship between Fine Motor Skills and Early Reading Acquisition

Several empirical studies have investigated the relationship between FMS and reading skills [26,27,28,29]. Although it could be surprising that FMS are associated to reading findings of Grissmer and colleagues on three large longitudinal studies indicate that FMS predicted fifth grade reading achievements after controlling for cognitive, gross motor, socioeconomic status, behavioral and social skills, general knowledge, and attention. One possible explanation that might account for this link could be that reading requires the use of several FMS to be displayed. Controlling eye movement for word tracking is needed to decipher the graphemes, mastering fine motor oral muscles is mandatory to enable the reader to turn letters into sounds and to show fluency with the code (reading words quickly), and guiding fine finger movements and eye-hand coordination is essential to write legibly. However, multidisciplinary and neuropsychological studies confirm that links between FMS and reading exist because primary brain regions are co-activated when doing certain motor or cognitive tasks. Conversely, many developmental disorders provide evidence for a prevalent co-occurrence of both motor and cognitive deficits [30]. Cameron and colleagues [31,32] investigated the contribution of FMS and executive functions to children achievement and improvement during the kindergarten. Authors found that design copy performance was associated with gains in literacy related domains (decoding, reading comprehension, and overall reading). Suggate and colleagues [33] examined experimentally the causal influence of graphomotor skills on decoding skills manipulating children’s writing condition. Findings demonstrate that children in the impaired writing condition learn to decode at a slower rate than their peers in the normal or pointing writing condition. Results provide evidence to the idea that graphomotor skills are important school readiness factors that support childhood learning and that FMS has a unique contribute to reading [34].

### 1.3. Relationship between Name Writing and Literacy

Literacy research has identified several predictors of writing and reading acquisition. Across three different domains (decoding, reading comprehension, and spelling and writing), name writing was reported in the National Early Literacy Panel [35] as one of the key precursors of children’s later literacy achievement even when controlling for IQ and SES. Although associated with knowledge of letter names and letter sounds, name writing is not merely linked with print-related skills, but with quality and production of written expression in kindergarten [36] and beyond [37].

Moreover, neuroscience studies have demonstrated that early writing plays a role in helping children to build code-related skills useful in encoding and decoding tasks [38]. Indeed, writing by hand has been found to support the stable memorization of letters’ form which later help children in learning to read [39]. Name production has been found to correlate with alphabet knowledge, word recognition, and concept of word for 4- and 5-year-olds, while name letters represent approximately 40% of children’s random-letter written characters [40]. Name and letter writing showed moderate association with later spelling in the NELP report [35]. Weinberger found that letter naming and letter writing at five were linked with reading skills assessed when children were 7 years old [41]. Since writing letters in one’s name may reflect skills involved in early literacy, examining the ability to write fluently one’s name appear to be a proxy of emergent writing [42].

### 1.4. Implication for Practice

The increasing body of evidence indicating that FMS influence academic success [43], student self-esteem, and cognitive development, suggests providing early activities and intervention programs to enhance FMS among kindergarten children. Given the results of studies linking FMS to mathematic reasoning [44,45], lexical processing [46], and reading skills [32,34] it would be desirable that future teaching and educational programs stimulate the development of FMS early, in order to ensure optimal cognitive development and fruitful academic success. Children who are weak on FMS might be disadvantaged in many educational domains such as mathematic and literacy, with the risk of widening the gap away from peers more able in using writing implements to write and handwrite [47,48,49,50]. Traditional service provision focuses on delivering interventions via referral to occupational or physical therapists in individualized sessions. Healthy staff is increasingly pressured by shortages, waiting lists are often overcrowded, inequity in the access to health support and poor coordination among services are reported as the most likely shortcomings of referral-based model assistance. On the other hand, parents report dissatisfaction with the provided health service support and would prefer a more immediate form of assistance, closely integrated with the child’s daily life and needs [51,52].

Searching for an inclusive solution, some studies have introduced occupational therapists (OTs) in the classrooms [53,54,55]. With the aim to provide targeted services, in small groups, to those children struggling in participating at the required classroom activities. Therapists working in an ecological context are integrated in the school team, collaborating with educators and providing timely and individualized support, thus promoting wellbeing and health in the school community. Closer collaboration across educational institutions and health services can facilitate prevention processes and more rapid diagnosis actions. Such approaches could be beneficial because treatments could be delivered within the routine classroom, more frequently and in inclusive situations supporting the effectiveness of motor skills interventions. Moreover, such integrated practices have also the advantage of sharing of experiences and skills across different professions, such as rehabilitation/healthy staff and educational teachers.

### 1.5. The Current Study

The objective of the present study was to verify the effectiveness and stability over time of possible changes occurring in first graders on the foundational skills predictive of writing success after a motor-perceptive training based on occupational therapy treatment interventions. For this purpose, the outcomes of the educational activities were explored through the assessments of children’ manual dexterity and visual-motor integration in three previously planned time-points: one at the baseline, at the beginning of the school year (T1), one after the intervention program (T2), and one at a distance of one month from the end of the upgrading activities (T3).

Based on previous experimental research our hypothesis was the following: that the intervention program would have a positive effect on the children’s fine motor skills and visual-motor integration at T2 compared to the baseline. We had no hypothesis about the stability over time of the effectiveness of the educational activities.

## 2. Materials and Methods

### 2.1. Participants Characteristics

This study is part of a longitudinal research program conducted at the Free University of Bolzano/Bozen. Data were collected from a convenient sample of first graders attending a school situated in the district of Bolzano, Northern Italy. The intervention to enhance fine motor skills was carried out with a class consisting of 13 students, 5 males and 8 females (age range in months 74–83, M = 78; DS = 2.73). The trained convenient sample consisted of typically developing children, with ordinary educational background (three years of kindergarten), attending regularly the first class. Educational special needs were not present at the time of the study.

The intervention study included educational activities for students of the all class. All data were collected by experienced researchers qualified in administration of psychological standard measurements. Parents of all students participating in the research study completed the informed parental permission forms. First graders whose parents provided written permission to participate to the study were asked to agree to be tested. The families, as well as the students, had the possibility to decline participation at any time from the research and were granted to ask questions. The data collected were treated according to the Privacy Act. Participants were included in the study if written consent was signed and if their medical history did not report any serious developmental or sensory deficiencies.

The intervention spanned 10 weeks, with two sessions per week. A total of 20 sessions were completed and each session lasted approximately 45 min. The intervention included educational activities enhancing fine motor skills and mastering eye-hand coordination abilities of 13 first graders with typical development.

The analysis of the sociodemographic characteristics of study’s participants included some family background variables (i.e., family income per year, educational level expressed in years of education, number of siblings, and age means). Overall, it emerges that parents had a medium-low income and families were quite numerous (33.3% had in fact more than three children almost three times the average number of children per Italian family; mean = 1.29). Mothers were more educated than fathers at the university level. Indeed, 25% of mothers and 9% of fathers reported to be university graduates. Inversely, 54.6% of the fathers and 41.6% of the mothers completed high school. The percentage of parents who had finished the middle school were very similar: 33.3% of mothers, and 36.4% of fathers (Table 1).

### 2.2. Procedure

The school was contacted by the research team to present the objectives of the study and explain the procedure. The study was organized in four phases: (a) baseline assessment of children’ fine-motor and visual-motor integrations skills (T1); (b) administration of the educational activities; (c) post-testing (T2); and (d) follow-up assessment one month after the post-testing phase (T3). Test administration at pre-intervention, post-intervention, and follow-up occurred during school hours, in an individualized setting by personnel trained in the use of the instruments in question. Pre- and post-test data were collected within a 2-week time frame immediately before and immediately after the intervention, for a total interval of 14 weeks. The time interval between post-test and follow-up was of 4 weeks. The administration of the assessment tools was accomplished by two trained psychologists in charge of carrying out the data collection, blind for the purposes of the study and condition (pre-test, post-test, and follow up) while scoring. Parents gave informed consent for the children’s participation to the study prior to research begin. The study was conducted in accordance with the Declaration of Helsinki, and the protocol was approved by the Ethics Committee of Faculty of Education of the Free University of Bozen-Bolzano (Project identification code: BW2057).

### 2.3. Instruments

*VMI (Visual-Motor Integration Test)*. The Beery–Buktenica Test for visuo-motor integration is a norm-referenced test to measure visual-motor integration in school-aged children. The tool was developed and standardized in the USA on a sample of over 11,000 children [56] in order to provide a rapid screening of learners having difficulty in coordinating visual-perceptual and motor information. The complete version of the VMI consists of 27 developmental geometric shapes organized in a progressive order of difficulty. The child is required to observe the different stimuli and copy the geometric forms with paper and pencil in a blank space. Two supplemental standardized tests, the Visual Perception and the Motor Coordination tasks, measure the visual discriminative skill to point the target figure (VMI-VP) and the ability to trace stimuli connecting dots within provided borders (VMI-MC), respectively. The raw VMI score is based on the number of correctly copied forms, accurately discriminated figure (VMI-VP), and properly drawn stimuli (VMI-MC). Standard norm-referenced scores are available for the Italian population [57]. The Beery-Buktenica VMI is a widely used tool which enjoys considerable psychometric validity, with high correlation coefficients for test-retest (*r* = 92) and inter-rater reliability (*r* = 93) and adequate construct validity [58]. Times required for completing the VMI vary from 15 to 20 min.

*Movement ABC-2 (Movement Assessment Battery for Children-2)*. The Movement ABC-2 is widely used standardized test for the identification of motor disorders in children aged between 3 and 16 years in many clinical and research settings [59]. The level of motor functioning of the child is ascertained through objective performances exhibited in 8 proposed tasks. The Movement ABC-2 is organized into three sections: (a) manual dexterity (MD); (b) to aim and grasp (AC); and (c) balance tasks (BAL). For the purpose of this study we administered only the three MD tasks assessing each child’s ability to use hands and manipulate objects. Children’ speed and accuracy were measured on posting coins into a piggy bank with one hand (MD1), in a timed bimanual assembling task of threading beads (MD2), and on drawing trail (MD3). The Movement ABC-2 has shown good psychometric properties with a reliability coefficient ranging from 0.73 to 0.84. Time required to complete the three Manual Dexterity tests is about 20 min.

*Name Writing Task.* Children were asked to write their own first name on a commercial touch-sensitive digitizer tablet (WACOM Intuos Pro and Intuos Inking Pen 4) connected to a notebook. The software handling the acquisition of handwriting movements was the package CSWin [60] installed in the computer. A blank sheet of paper and a regular inking pen were placed on the surface of the digitizer providing the impression of a conventional writing condition. While subjects were writing on the blank sheet receiving a visual feedback of their products and a haptic feedback of the normal pen’s friction on the surface, the tablet had recorded the written traces. Position of the pen tip is registered with a spatial resolution of 0.5 mm and a temporal resolution of 200 Hz with accuracy of 0.1 mm. The inductive measuring method allows to record handwriting movements even if the stylus is lifted (up to 1 cm) above the digitizer surface [61].

Kinematic variables used to investigate handwriting movements were frequency of strokes (FREQ) measured in Hertz and stroke pressure (PRESS) assessed in Newton and automaticity expressed in number of inversion of velocity (NIV). Handwriting speed was measured as frequency (FREQ) of upward and downward movements in 1 s recorded by the CSWin [60]. Mai and Marquardt report that previous studies with adults showed average stroke frequencies of around 5 Hz [62]. This measure seems to be more reliable than stroke length per second (mm/s), which depends on a person’s writing size. PRESS refers to the average amount of the pen’s tip pressure on the paper surface normally ranging between 1 and 1.5 Newton in adults without neurocognitive deficiencies [61]. Automaticity of handwriting movements was rated in terms of number of inversions of velocity (NIV), which indicates the number of directional changes in movement execution. When handwriting is fully automated requires only one velocity change per stroke. Indeed, highly skilled, fluent writers produce smooth and automatic handwriting movements with acceleration/deceleration ratio equal to one [63].

### 2.4. Intervention Program (Educational Activities)

The intervention program was developed for this study by the first author and a team of occupational therapists working at the “Claudiana” Medical School of Health Professions to promote foundational skills for handwriting. The educational activities consisted of short games to be carried out in a small group, structured according to the principles of occupational therapy, but adapted to the school context. All activities aimed to stimulate children’s fine motor skills and the ability to coordinate visual information with graphic performance requirements. The games involved practice with in-hand manipulation, transfer of objects from the palm of the hand to the fingers, dissociation and coordination of fingers’ use, discrimination of forms, figure-background separation and completion of paths and tracks. A total of 7 games were proposed to children in three level of difficulty (low, medium, high) for a total of 21 variants. In each intervention session three groups played at the same time three different educational activities and in rotation the occupational therapists administered the different activities and variations so that each group could experience each activity in its three different levels of difficulty. As an example, to complete the game “Tutti Frutti” (i.e., “All fruits”) children had to carry out different activities according to the request pictured in the card drawn in correspondence with the box where their pawn was to be found. Activities required the use of commonly used and easily available material such as scissors, glue, pencils and markers of different thickness, woolen and twine threads, tissue paper, pipe cleaners, colored paper and cardboard of various thickness, small spheres of different size and weight, raisins, peanuts, rice, and pasta for soups in the shape of a letter of the alphabet.

Intervention sessions occurred two times/week from October to December, following the school calendar. Each session lasted approximately one hour for a total of two hours per week. The overall duration of the intervention (TOT = 20 h) in the present study is slightly higher than the median (M_edian_ = 16 h) of the duration of the interventions reviewed by Eddy and colleagues [24]. Interventions were conducted from two occupational therapists with the support of two classroom teachers. The script of each session was as follows: (1) the class was organized in three small groups and activity of the day was introduced and explained to each small group by occupational therapists and teachers; (2) children played the games practicing the activities with support and aids from the educational team; and (3) each session ended by putting a stamp in a card board prepared for the children where they could see the progress of the work and the program.

### 2.5. Statistical Analysis Plan

Univariate analysis of one-way repeated-measure ANOVAs were performed to verify if there were significant differences in first graders’ performance on visuo-motor integration (VMI) and fine motor skills tests (MABC-2) at three time points (pre-test, post-test and follow-up). No outliers were detected, as assessed by the boxplot inspection for values greater than the 1.5 box-lengths from the edge of the box. Performance scores were normally distributed at each time point (T1, T2, and T3), as shown by Shapiro-Wilks test (*p* > 0.5 for VMI and its Supplemental tests, and for the Manual Dexterity scale of the MABC-2). Moreover, Pearson product-moment correlations were run to determine the strength and direction of linear relationships between dynamic writing performance variables (frequency, pressure, and automaticity) and children’s raw scores in visual-motor integration tests and fine motor skills before and after the intervention program and at the follow-up time points.

## 3. Results

**Figure 1:**
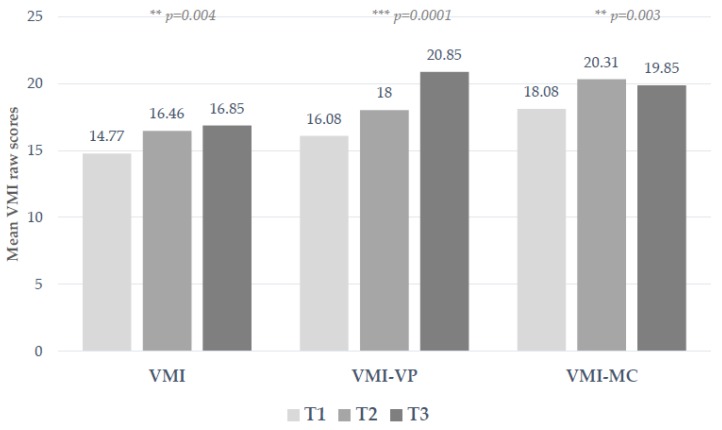
Mean differences in children’s visual-motor integration performance scores (VMI), discrimination abilities (VMI-VP), and motor coordination (VMI-MC), measured at baseline (T1), after the intervention program (T2), and at follow-up (T3). POST-HOC (Bonferroni). VMI: T2 > T1 *p* = 0.04; T3 > T1 *p* = 0.02; VMI-VP: T3 > T1 *p* = 0.003; T3 > T2 *p* = 0.01; VMI-MC: T2 > T1 *p* = 0.008; T3 > T1 *p* = 0.02. ** *p* < 0.01, *** *p* < 0.001.

Mauchly’s test indicated that assumption of sphericity had not been violated for any of the included dependent variables (χ^2^ VMI _(2)_ = 1.503, *p* = 0.472; χ^2^ VMI-VP _(2)_ = 1.906, *p* = 0.863; χ^2^ VMI-MC _(2)_ = 0.436, *p* = 0.804; χ^2^ MD _(2)_ = 2.554, *p* = 0.279). the children’s visuo-motor integration was statistically significantly different (Figure 1) at the three time points during the intervention program increasing remarkably from pre-test, to post-test and follow-up (F _(2.24)_ = 6.936, *p* = 0.004, partial ω^2^ = 0.366). The proposed activities elicit an increase in the performance of children in this area from T1 (M = 14.77; SD = 2.83) to T2 (M = 16.46; SD = 2.47) to T3 (M = 16.85; SD = 2.82). The significant change concerns the comparison between children’s performances between pre- and post-intervention, and between pre-test and follow-up, but not from T2 to T3. The visual discrimination scores also increase (F = _(2.24)_ = 10.977, *p* = 0.0001, ω^2^ = 0.478). Post-hoc analysis with Bonferroni correction shows that the ability to distinguish and recognize forms increases over time reaching levels of significance between T1 and T3 (M = 16.08; SD = 4.09; M = 20.85; SD = 3.21) and between T2 and T3 (M = 18.00; SD = 3.16; M = 20.85; SD = 3.21), but not between T1 and T2. Graphomotor performance significantly changes over time (F _(2.24)_ = 7.641, *p* = 0.003, partial ω^2^ = 0.389), between T2 and T1 (M = 20.31, SD = 2.86; M = 18.08; SD = 3.12), and between pre-test and follow-up (M = 18.08; SD = 3.12; M = 19.85; SD = 2.44). The manual dexterity scale of the MABC-2 does not improve significantly over time (F _(2.24)_ = 1.336, *p* = 0.282, partial ω^2^ = 0.100), although the timed bimanual task of threading beads is affected by the intervention program and children reduce significantly the time to complete this task (F _(2.24)_ = 10.477, *p* = 0.001, partial ω^2^ = 0.466).

Pearson’s correlations between children’s scores on the VMI and Movement ABC-2 tests and the handwriting performances measured with tablet digitizer are reported in Table 2. At T1 visual motor integration raw scores were significantly negatively associated with NWT-FREQ (T1: *r*_(13)_ = −0.69, *p* = 0.008), while at T2 and T3 VMI performances weren’t significantly related with children’s frequency in the Name Writing Test NWT-FREQ (T2: *r*_(13)_ = 0.03, *p* = 0.20; T3: *r*_(13)_ = 0.37, *p* = 0.91). From baseline to after intervention the sign of the correlation coefficient related to the frequency of written traces changes from negative to positive and remains positive at the follow-up assessments for the visual discrimination abilities and for the fine motor control skills.

The frequency of writing was found to be statistically negatively related to automaticity at the three time points (T1: *r*_(13)_ = −0.71, *p* = 0.006; T2: *r*_(13)_ = −0.76, *p* = 0.002; T3: *r*_(13)_ = −0.85, *p* = 0.0001). The magnitude of the correlation between writing speed and fluidity of the movement was strong and explained between 50% to 72% of the variation in automaticity. Pen pressure (PRESS) was negatively associated with visual motor coordination and manual dexterity from T1 to T3 but at follow-up the relationship is statistically significant (T3: *r*_(13)_ = −0.60, *p* = 0.02). A statistically significant association has been found between pen pressure and automaticity only at T3 (T3: *r*_(13)_ = 0.68, *p* = 0.01).

A series of within-subjects ANOVA’s were run to determine whether there were any statistically significant differences between the means of temporal parameters of the Name Writing Test at T1, T2, and T3. The assumption of sphericity was met, as assessed by Mauchly’s test of sphericity (χ^2^ FREQ_(2)_ = 2.887, *p* = 0.236; χ^2^ PRESS_(2)_ = 5.771, *p* = 0.056; χ^2^ AUTO_(2)_ = 0.106, *p* = 0.949). No significant effect of time on writing fluency (F_FREQ_ = _(2.24)_ = 2.197, *p* = 0.133, ω^2^ = 0.155), pen pressure (F_PRESS_ = _(2.24)_ = 1.790, *p* = 0.190, ω^2^ = 0.129), and automaticity (F_AUTO_ = _(2.24)_ = 2.098, *p* = 0.145, ω^2^ = 0.149) was detected. The psychoeducational intervention did not elicit statistically significant changes in handwriting skills over time. Descriptive statistics of the main study’s variables are reported in Table 3 as means and standard deviations at the three assessments time points.

## 4. Discussion

This study aimed to verify how the visual motor integration and manual dexterity of first graders, who underwent a graphomotor intervention program, vary over time and to investigate possible changes in the kinematic parameters describing handwriting. Written traces acquired by means of the CSWin software have been analyzed and examined with respect to their relationship with handwriting foundational skills. Participants were tested three times: at baseline, i.e., before the educational activities, after the 10-weeks lasting training, and at the follow-up one month later. Overall, results show significant changes in children’s visual-motor integration scores and graphomotor abilities along the intervention program. Moreover, visual discrimination abilities increase significantly over time from the baseline to the end of the study but without reaching significant differences between pre- and post-intervention, eliciting some interesting reflections with respect to the different growth trends of the various skills examined.

Firstly, it should be noted that visuo-motor integration, visual discrimination, and graphomotor skills do not develop longitudinally in the same way. The ability to coordinate in efficient pattern handwriting movements and visual information to copy geometrical stimuli increases continuously from the baseline to the end of the research study, but the advance in performance is much higher between pre-test and post-test than between post-test and follow-up. This important developmental skill linked with many functional academic abilities required to participate to school daily life and later achievements [64] benefits from the interventional program raising significantly from T1 to T2. Recently, Wicki and Hurschler [65] found that visual-motor integration skill predicts orthographic ability in fourth graders, drawing attention to this link and stressing the need to further clarify the mechanism underlying this association. In any case, educational activities promoting visual-motor integration skills in an ecological setting (and not in an individualized or clinical one) indirectly enhance the learning of orthography. Previous studies have namely identified VMI as a predictor of reading and writing success [11,14], and revealed that is associated with text legibility [66,67]. Further research is needed to understand the generalizability of this finding and the possible variability at different educational grades or in different target samples. It appears plausible that visual-motor integration skills are likely to have more weight in the acquisition of writing automaticity and in the learning of orthography in the first grades of education than in the following years.

Children of this study were asked to write by hand their own name on a digitizer that recorded their pen activity. Handwriting skills of study’s participants were assessed analyzing pen movement frequency, pressure on the digitizer surface, and writing automaticity. Findings reveal no significant changes over time on kinematic variables used to investigate children’s ability to write their own names. In contrast with our expectations we could not demonstrate an intervention effect on temporal characteristic of first graders transcription skills. A possible explanation for the missing significant changes over time is the fact that the writing tasks presented might be already quite automated. Children learn to write their name well before they know the letters of the alphabet and learn to write them. If on the one hand the task of writing the own name had the advantage of not requiring any literacy, on the other it was a task in which the children certainly already had developed a certain fluency. It would probably have been more appropriate investigating the temporal changes in transcription skills while tracing forms, symbols or letters less familiar because they were not included in children’s names. However, besides the fact that the intervention did not yield differential effects on fluency, pressure and automaticity, further qualitative analyzes shed new light on the changes that have taken place in this group of first graders over time.

Correlational analyses on kinematic parameters and visual motor integration skills along the three time points showed that the frequency of upward and downward strokes changes sign from the beginning to the end of the study. At baseline writing frequency was negatively associated with the ability to integrate visual information and finger-hand movements. In the following assessment time points the association became positive as reported in other studies [65,68]. A possible explanation may be that educational activities have drawn children attention to the name writing task, so that in the second and third test children show to be more conscious of the way in which letters must be written. Hence, an increase in the number of oscillation stroke’s may be related to a greater accuracy in writing.

Children’s writing frequency has been found to be significantly negatively associated with writing automaticity. This finding describes the fluency in handwriting in terms of relationship between number of acceleration and deceleration and mean frequency in upward and downward movements. Fluent handwriting presents a ratio between accelerations and decelerations equal to 1, and the amount of upward and downward movements equal to 5 in adults [60]. It is interesting to note not only that this relationship remains stable over time but also that the magnitude of correlational coefficients increases in the post-intervention and further develop in the follow-up. Previous studies have shown the link between automaticity and writing speed (measured in number of words in a defined time) concluding that the ability to write fast is influenced by the automation of hand and finger movements [65]. However, considering frequency and then assessing speed as stroke length per second (mm/s) seems to be more appropriate since less dependent from a person’s writing size.

Finally, we observed a lack of statistically significant association between manual dexterity scores and automaticity. A possible explanation could be that the name writing test involved the cognitive dimension related to letter knowledge and to the motor sequence required for their completion rather than the coordination of the small hand muscles. This result supports the idea that a kinematic analysis of handwriting allows a refined exploration of quantitative evaluation parameters for writing performance (pauses, movements of the pen’s tip in the air, accelerations and decelerations in drawing strokes) that can give descriptive diagnostic insights into handwriting difficulties and provide valuable indications for their treatments [69,70]. Indeed, recent studies on temporal characteristics of handwriting on clinical samples have demonstrated that children with developmental dyslexia are slower in producing letters, make significantly more pauses, and show less legible handwriting [71]. Results indicate that using a digitizer may help to analyze the course of handwriting and may be highly relevant and useful for practitioners to set resources and interventions on handwriting.

This research has several limitations that lead to suggestions for future investigations. Although the effectiveness of this training cannot be demonstrated according to the golden standard rules of empirical research (due to the lack of a control group and a randomization), as often happens in educational studies, future research may adopt some measures that improve the understanding of how much this intervention supports the development of children in the areas of interest. Additional investigations may ascertain if the improvements on visual-motor abilities in this pilot study are due solely to the stimulation of psychoeducational activities using a two-group non-randomized controlled trial with pretest-posttest design. To conclude that any change that might be found in the treatment group would be associated to the intervention rather than to other factors, baseline characteristics of the two groups should be tested before starting with the research (i.e., children’s visual acuity, executive functions, and motor development). Failure in matching experimental and control groups could threaten the validity of the study and make it impossible to draw meaningful conclusions.

Besides, the link between fine motor skills and handwriting needs to be further explored. The lack of association between motor competence in the coordinated use of hands and fingers and kinematic analysis of the strokes suggests that the graphic transcription process is strongly influenced by a cognitive component attributable to letter’s knowledge and the recovery from memory of the motor sequence necessary for their completion. Moreover, longitudinal research would be desirable to examine long-term effects of this intervention program to establish whether there are later impacts on writing performance.

A final issue concerns the intervention program itself. The fact that no significant changes occur over time in manual dexterity scores should lead us to introduce some changes in the proposed psychoeducational activities. It is plausible that the games, although arranged and graded in three difficulty levels, would be not sufficiently differentiated from the already acquired children’s skills and hence were not able to promote significant improvements. In this case it may be appropriate to adjust the games and differently grade the difficulties for each trained skill. Another possible explanation of the missing gains on fine motor abilities could be the difficulty in training children’s skills in a group situation. Activities were conducted from trained occupational therapists who usually work in individual clinical setting and were not familiar with classroom context, as it is not generally used and expected in Italian schools that clinical professionals contribute to educational curriculum and didactic activities. The ability of the occupational therapists involved in our study to control how children displayed and executed hand and finger movements could have been reduced in the group condition. It would be congruent to trainee occupational therapists to work in ecological situations such as schools with the aim to prevent motor difficulties and promote learning to write. Moreover, it is possible that a program lasting longer than 10 weeks including more training unites can enhance handwriting skills among first graders.

From a general point of view, future research should address also the qualitative analyses of the letter production because learning to write by hand requires learners to tackle a double aim: increasing the fluency of producing written traces and improving the legibility of the handwritten text. In contrast with the assumption that gains in motor control would immediately translate into greater fluency, we might suppose that first graders the more they understand that letters need to be well formed to be legible the more they will pay attention to the formal quality of letters to preserve their communicative function at the expense of handwriting fluency. In this case, a qualitative analysis of the letter production should supplement the quantitative temporal analysis of handwriting allowing a greater knowledge on learning processes and motor control during emergent literacy.

Finally, we used a small convenience sample to test the power of psychoeducational activities. A larger, more representative sample is needed to proof our findings and provide generalizable results in typically developing children or in individuals with clinical conditions such as dysgraphia or developmental coordination disorder. Furthermore, future research on kinematic parameters of handwriting are needed to promote our understanding of the link between foundational skills required to perform graphic traces and the acquisition of a fluent writing in early academic grades.

## Figures and Tables

**Table 1:** Descriptive statistics of the study participants expressed in absolute values, percentages, means and standard deviations (SD).

Demographic Variables	N	%
Gender		
female	8	61.5
male	5	38.5
Handedness		
left-handed	1	7.7
right-handed	12	92.3
Educational Level Mother in years		
middle school (0–8y)	4	33.3
high school (9–13y)	5	41.6
university (14–18y)	3	25.1
postgraduate education (after 19y)		
Mother’s age (Mean; SD)	39.42	4.87
Educational Level Father in years		
middle school (0–8y)	4	36.4
high school (9–13y)	6	54.6
university (14–18y)	1	9
postgraduate education (after 19y)		
Father’s age (Mean; SD)	42.50	5.83
Family Income per year		
low (<35000 €)	9	75
medium (between 36000 and 50000)	3	25
high (>51000)		
Number of siblings		
1	3	25
2	5	41.7
3	2	16.7
4 and >4	2	16.6

**Table 2:** Pearson’s correlations between Writing Test kinematic parameters and children’s visual-motor integration and manual dexterity scores at T1, T2, T3.

	VMI	VMI VP	VMIMC	MD	NWT FREQ	NWT PRESS	NWT AUTO	VMI	VMIVP	VMIMC	MD	NWT FREQ	NWT PRESS	NWT AUTO	VMI	VMI VP	VMI MC	MD	NWT FREQ	NWT PRESS	NWT AUTO
	T1							T2							T3						
VMI	1	0.62 *	0.38	0.46	−0.69 **	−0.31	0.55 *	1	0.40	0.48	0.30	0.03	−0.47	−0.03	1	0.47	0.58 *	0.26	0.37	−0.60 *	−0.29
VMI-VP		1	0.48	0.37	−0.15	−0.12	0.07		1	0.59 *	0.33	0.39	−0.15	−0.29		1	0.87 ***	0.03	0.34	−0.21	−0.32
VMI-MC			1	0.13	−0.32	−0.31	0.26			1	0.43	0.15	−0.51	−0.18			1	0.13	0.51	−0.50	−0.59 *
MD				1	−0.19	−0.45	0.35				1	−0.01	−0.48	0.15				1	0.09	−0.31	−0.11
NWT FREQ					1	0.17	−0.71 **					1	0.12	−0.76 **					1	−0.44	−0.85 ***
NWT PRESS						1	−0.13						1	0.02						1	0.68 **
NWT AUTO							1							1							1

Note: VMI: Visual-Motor Integration; VMI-VP: Supplemental Test of Visual Perception; VMI-MC: Supplemental Test of Motor Coordination; MD: Manual Dexterity Scale of the Movement ABC-2 (MD1 posting coins, MD2 threading beads, MD3 drawing trails); NWT-FREQ: Name Writing Test Frequency (Hertz); NWT-PRESS: Name Writing Test Pressure (N); NWT-AUTO: Name Writing Test Automaticity (NIV). * *p* < 0.05 ** *p* < 0.01 *** *p* < 0.001.

**Table 3:** Descriptive statistics of children performances on Beery Buktenica Visual Motor Integration Test, Fine Motor Tasks of the Movement ABC-2, and Name Writing Test performed on a digitizer. Data are presented as means and standard deviations of the children’s raw scores.

	T1		T2		T3	
	M	DS	M	DS	M	DS
VMI	14.77	2.83	16.46	2.47	16.85	2.82
VMI-VP	16.08	4.09	18.00	3.16	20.85	3.21
VMI-MC	18.08	3.12	20.31	2.86	19.85	2.44
MD1-PH	16.62	1.60	17.46	2.53	18.54	4.99
MD1-OH	19.23	2.45	22.92	5.88	22.00	6.59
MD-2	40.62	7.71	35.31	10.55	32.15	8.27
MD-3	0.62	1.19	0.62	0.65	0.31	0.63
NWT FREQ	1.32	0.52	1.48	0.79	1.68	0.76
NWT PRESS	1.88	0.72	1.73	0.72	1.60	0.69
NWT AUTO	3.44	1.92	3.69	2.49	2.59	1.25

Note: VMI: Visual-Motor Integration; VMI-VP: Supplemental Test of Visual Perception; VMI-MC: Supplemental Test of Motor Coordination; MD1-PH: Manual Dexterity Scale 1—Preferred Hand (MD1 = posting coins); MD1-OH: Manual Dexterity Scale 1—Other Hand (MD1 = posting coins); MD2: Manual Dexterity Scale 2 (MD2 = threading beads); MD3: Manual Dexterity Scale 3 (MD3 = drawing trails); NWT-FREQ: Name Writing Test Frequency (Hertz); NWT-PRESS: Name Writing Test Pressure (N); NWT-AUTO: Name Writing Test Automaticity (NIV).
